# Jaw relation recording using nick and notch technique with intraoral gothic arch tracing in completely edentulous patients: A clinical study

**DOI:** 10.6026/973206300222521

**Published:** 2026-04-30

**Authors:** Arvind Kumar Singh, Shruti Sharma Grover, Nivedita Rai, Vunshree Gupta, Chhavi Sharma, Satya Prakash Gupta, Satyavrat Singh

**Affiliations:** 1 Department of Prosthodontics Chandra Dental College & Hospital, Barabanki, India; 2 Department of Prosthodontics, Private Practitioner, Lucknow, Uttar Pradesh, India; 3 Department of Oral and Maxillofacial Surgery, Chandra Dental College and Hospital, Barabanki, India

**Keywords:** Centric relation, gothic arch tracing, nick and notch, split-cast mounting, edentulous patients, jaw relation

## Abstract

Recording centric and protrusive jaw relations in completely edentulous patients remain a critical yet challenging step in complete denture fabrication. Therefore, it is of interest to compare the consistency of jaw relation records obtained using Nick and Notch, intraoral Gothic arch tracing and extraoral Gothic arch tracing methods with split-cast mounting on a semi-adjustable articulator. Hence, twenty edentulous subjects (45-60 years) were evaluated and discrepancies were measured at right lateral, left lateral and anterior points using a digital Vernier caliper. Statistical analysis using one-way ANOVA and Tukey's post hoc test demonstrated that the Nick and Notch technique exhibited the least discrepancy, while extraoral Gothic arch tracing showed the highest inconsistency, with intraoral tracing demonstrating intermediate values. The Nick and Notch technique was found to provide the most reliable jaw relation records, with intraoral Gothic arch tracing being more accurate than extraoral tracing, likely due to reduced muscular interference and closer approximation to the condylar rotational axis.

## Background:

The transfer of maxillomandibular relationship between the patient and the articulator is critical in the successful rehabilitation of entirely edentulous patients with complete dentures. The registration of centric relation may be considered one of the most important steps in the process of complete denture construction as any errors made at this stage spread over to all other stages and may eventually result in the destruction of the entire functional and comfort levels of the prosthesis as well as its lifespan [1, 2]. The maxillomandibular relation has also been described as centric relation, whereby the condyles line up with the thinnest avascular structure of their respective discs which lie in the most anterior-superior position in respect to the posterior slopes of the articular eminences [3]. This posture has a clinical importance as it is a reproducible, bone-braced reference posture when all the movements of the mandible start and end when swallowing and in habitually closing the mouth [4]. Proprioceptive information of the periodontal ligament offers accurate control of mandibular positioning during functional movements in dentulous individuals. This proprioceptive system is lost entirely by the loss of the natural dentition and the sense direction is handed over to the temporomandibular joint largely to the musculature which surrounds it [5]. This basic change in the sensory feedback makes the correct identification of centric relation in the edentulous patients much more difficult than the dentate counterparts. To make the matter even more challenging, long-term edentulism, chronic closure behavior, neuromuscular impairment and temporomandibular disorders can make the clinician less effective when it comes to positioning the mandible in a consistent centric position [6, 7]. Various methods have been created and promoted to record centric relation and they are broadly categorized as either being a static method or a functional method [8]. The method known as the Nick and Notch wax registration technique, which is a form of the static method, is where the mandible is positioned in centric relation and then the relationships between the opposing occlusal rims are recorded via an interocclusal recording medium. These techniques are appreciated due to their ease, low base displacement and dependence on the natural habitual pattern of closure of the patient [9]. Functional methods on the other hand, have active mandibular movements during recording and include the different graphic tracing techniques. The Gothic arch tracing (by use of intraoral or extraoral tracing devices) is one of the most frequently used graphic techniques of establishing centric relation in edentulous patients among functional techniques [10, 11]. The Gothic arch tracing method is based on the fact that when the mandible performs lateral excursive movements of the retruded position, the tracing stylus will trace an arrow-shaped mark on the recording plate and the apex of the arrowhead will be the centric relation position [12].

The intraoral tracing device places the recording apparatus in the oral cavity in the close proximity of the condylar axis of rotation as compared to the extraoral device which projects the recording platform forward of the oral cavity. According to proponents of the intraoral technique, its positioning closer to the condylar axis minimizes the amplification of error and that the oscillatory action of the oral muscles is relatively ineffective during recording [13, 14]. The proponents of the extraoral method argue that the wider tracing platform helps to more easily identify the apex and patient guidance that is more accurate [15]. Although these techniques have been widely used clinically over the past decades, there is little comparative information assessing the comparative accuracy and stability of the three popular techniques in standardized conditions under objective quantitative evaluation. There have been past studies comparing the selected pairs of these methods, although three-way comparisons with split-cast mounting used as the standard of verification are few and far between in the literature of today [16, 17]. Split-cast mounting technique was first introduced by Needles, which offers an accurate and repeatable way of assessing the accuracy of interocclusal records by determining the difference in the split surfaces between records on different occasions placed on top of each other [18]. Therefore, it is of interest to assess and compare of the relative consistency of centric and protrusive jaw relation records recorded with using Nick and Notch method, intraoral Gothic arch tracing and extraoral Gothic arch tracing methods in entirely edentulous patients, analyzed in terms of split-cast mounting on a semi-adjustable articulator.

## Materials and Methods:

## Study design and setting:

The research was a clinical comparative study that was undertaken in the Prosthodontics Department of a dental teaching institution within a 6-month period.

## Population of the study and criteria of selection:

The outpatient department was used to select the twenty entirely edentulous patients using the stratified random sampling technique. Inclusion criteria included fully edentulous individuals aged 45 to 60 years old of both sexes, enough mouth opening, good residual ridges, absence of starting or acquired maxillofacial deformities and willingness to sign informed consent. Patients who had compromised systemic health, temporomandibular joint disorders, had severely resorbed ridges, were unable to open their mouths or did not want to do so were excluded.

## Sample size and grouping:

The patients acted as their controls and centric and protrusive jaw relationship was recorded through all the three methods producing a total of 60 records. The recording was clustered into the following:

Common Group (NN): Nick and Notch technique (n = 20)

The needlestick: NE (n = 20) Group I: Extraoral Gothic arch tracing.

Group II (NI): Intraoral Gothic teeth tracing (n = 20)

## Primary prosthodontic surgeries:

All patients were provided with standard procedures of prosthodontics. Impression compound in stock trays was used to get primary impressions and dental plaster used to cast primary impressions. Acylic resin self-curing trays with the border-molded using the green stick compound and eventual impressions on zinc oxide eugenol paste were made. Dental stone casts of the masters were prepared and acrylic resin record bases that were heat-cured and had wax occlusion rims were made. In order to facilitate standardization, plaster index method was employed in duplicating the record base of every patient to produce three identical record bases per patient of the three techniques of recording. Index duplication was equally used to standardize occlusion rims.

## Face-Bow transfer and mounting:

A random face-bow capture of each patient was made and sent to a Hanau Wide-Vue semi-adjustable articulator. The face-bow record was used to hold the maxillary cast on the articulator. The first mandibular cast mounting was done by using the Nick and Notch centric record which was used as the reference mounting throughout the rest of the tests.

## Recording techniques:

## Nick and notch method:

Two to three millimeters of the wax were used to take away on both sides of the mandibular occlusion rim at the premolar area and the surface was grooved. They were lubricated and corresponding V-shaped notches and nicks were made on the maxillary rim. The patient was instructed to close at the retruded position and zinc oxide eugenol paste was applied at the mandibular troughs to record the relationship. Recording was done five times and the most repeated record was chosen.

## Extraoral Gothic arch tracing:

To fix the upper bearing plate, the rim of the maxillary aligned with the occlusal plane. They were reduced by 3 mm on the mandibular rim and the lower tracing platform was connected. The tracing table was painted with lamp black. Centric and lateral excursive movements were instructed up until a definite Gothic arch pattern with a definite apex was achieved. A sheet of transparent plastic was perforated and placed on top of the apex with the centric and protrusive positions recorded with the type III dental stone between the rims.

## Intraoral Gothic arch tracing:

After decreasing the mandibular rim height by 3 mm, the intraoral tracing device was positioned to the occlusion rims. The bearing pin was then pushed until it made contact with the upper plate at the right vertical dimension. The permanent marker ink was used to cover the upper plate. The patient was made to do eccentric movements until a clean trace that showed a definite apex was achieved. It was perforated with a transparent sheet at the apex and centric and protrusive records were then made using type III dental stone.

## Split-cast evaluation:

After the Nick and Notch record mounting was done, the upper cast was separated with orthodontic plaster off the mounting disc. On the upper split cast three reference points were set that were an anterior point in front of the incisive papilla and two posterolateral points at the level of 10 mm in front of the posterior border of each maxillary tuberosity. The interocclusal records of each of the techniques were interposed sequentially between the mounted casts, the articulator shut and the difference recorded at each reference point using a digital Vernier caliper set to 0.01 mm. centric and protrusive positions were measured.

## Outcome measures:

Each technique had three measurements of discrepancy at centric position and protrusive position:

[1] Right lateral discrepancy (RLD)

[2] There is also lateral discrepancy that occurs to the left (LLD).

[3] Anterior discrepancy (AD)

[4] General disparity (average of RLD, LLD and AD)

## Statistical analysis:

The statistical evaluation was done using SPSS 22.0. To verify normality, Shapiro-Wilk test was used and Levene test was used to check homogeneity of variance. Mean, Standard deviation as a ratio was used to represent the descriptive statistics. Intergroup analysis was carried out through one-way analysis of variance (ANOVA) and then Tukey test, the honestly significantly different post hoc test was used to carry out a pair-wise analysis. The significant level was established at p < 0.05.

## Results:

The mean discrepancy values at the centric position for all three techniques across the three measurement sites are presented in Table 1 (see PDF). At all measurement sites, the Nick and Notch method demonstrated the smallest mean discrepancy, while the extraoral Gothic arch tracing exhibited the largest. The intraoral Gothic arch tracing produced intermediate values. One-way ANOVA revealed highly significant differences among the three groups for RLD (F = 162.50, p < 0.001), LLD (F = 83.27, p < 0.001) and AD (F = 91.21, p < 0.001). The overall mean discrepancy at centric was 0.15 ± 0.03 mm for Nick and Notch, 0.42 ± 0.10 mm for intraoral tracing and 0.74 ± 0.12 mm for extraoral tracing (F = 200.90, p < 0.001). The mean discrepancy values at the protrusive position followed a similar pattern (Table 2 - see PDF). The Nick and Notch method consistently produced the smallest discrepancies, while extraoral tracing demonstrated the largest values at all measurement sites. ANOVA revealed significant differences for RLD (F = 99.94, p < 0.001), LLD (F = 53.13, p < 0.001) and AD (F = 53.20, p < 0.001). The overall mean discrepancy at protrusive was 0.23 ± 0.08 mm for Nick and Notch, 0.51 ± 0.13 mm for intraoral tracing and 0.86 ± 0.14 mm for extraoral tracing (F = 146.70, p < 0.001). Tukey's post-hoc analysis confirmed that all pairwise differences were statistically significant at both centric and protrusive positions (Table 3 - see PDF). The extraoral tracing demonstrated significantly higher overall discrepancy compared to both intraoral tracing and Nick and Notch at both jaw positions. Intraoral tracing showed significantly higher discrepancy than Nick and Notch but significantly lower discrepancy than extraoral tracing at all measurement sites.

## Discussion:

This study has clearly shown that the records of the jaw relation show statistically significant hierarchical variation among the three methods which were evaluated, with the Nick and Notch technique yielding the smallest inconsistency, then the intraoral Gothic arch tracing and extraoral Gothic arch was tracing recording the highest inconsistency with the reference mounting in centric and protrusive positions. The implications of these findings are significant in clinical areas of selecting jaw relation recording methods in complete dentures fabrication. The high consistency of the Nick and Notch method, which could be seen in the current study, could be explained by several advantages that the static recording approach has. In contrast to graphic tracing techniques, which involve active mandibular excursive movements in recording the relationship, the Nick and Notch technique records the relationship of the jaws in a closed position in which the jaw is stationary and in a natural and habitual closure pattern of the patient [19, 20]. Lack of functional mandibular movements during recording reduces the lateral and anteroposterior movement of record bases relative to the supporting ridges, a phenomenon which has been recognized to be a major source of error in the functional recording techniques [21]. Also, because zinc oxide eugenol paste is used as the registration medium, it offers a firm and dimensionally stable record that is not distorted during transfer to the articulator [22]. The fact that intraoral Gothic arch tracing registered much less discrepancy compared to extraoral technique is not a novel finding in relation to the laws of biomechanics and supports prior clinical studies. The intraoral tracing apparatus aligns the central point of bearing and recording plate to the oral cavity much closer to the transverse axis of the hinge of the mandible [23, 24].

This geometrical advantage implies that any angular movement of the condylar region creates a correspondingly less linear movement at the recording plate than it would with the extraoral device where the tracing platform is several centimeters forward of the oral cavity. It has been identified that this effect of magnification is a root cause of inaccuracy of extraoral tracing systems [25]. Moreover, the smaller size of the intraoral apparatus also allows the oral musculature to be in a more relaxed physiological position during tracing, compared with the large extraoral equipment, which can cause an observable change in the neuromuscular behaviour and tongue positioning, which invalidates the mandibular guidance [26, 27]. The larger error found with extraoral tracing, even though its use is common in clinical practice, also could be explained by the fact that the assembling of the device and achieving consistent fixation of the extraoral parts to the occlusion rims during recording are not as simple. Tilting or displacement of the record bases can be caused by weight and leverage of the anteriorly extending tracing platform, especially in patients who have resorbed ridges and who already have weak bases [28]. Earlier studies that have compared the use of graphic and wax-based registration modalities have come to a conclusion that although the methods used in graphic approach are theoretically accurate, they are more technique-sensitive and are prone to errors due to non-stability of the device and patient compliance [29, 30]. The fact that the value of difference at the protrusive position is always higher than that of centric position in all three methods is also not surprising and is a characteristic of the fact that the recording of the eccentric jaw positions is more complex in nature. Protrusive registration involves bilateral condylar translation of the articular eminences, which adds further variables to the form of the articular disc-condyles, disc- condyle-relationships and viscoelastic behaviour of the retrodiscal tissues [31]. Any difference in the direction of condylar translation or lack of consistency in the extent of protrusion between recording tries would be exaggerated at the interface of the level of the occlusion rims and would result in more significant discrepancy at the split cast-interface [32]. Magnitudes of discrepancies noted in the study hold clinical value which should be taken into consideration. The total mean error of the Nick and Notch method had a range of between 0.15 mm to 0.23 mm at centric and protrusive respectively which is quite within the scope of what can be classified as a clinically acceptable set of values in complete denture fabrication. Mean discrepancies of 0.42 mm and 0.51 mm at centric and protrusive respectively were obtained using the intraoral tracing technique which, whilst statistically different to the Nick and Notch method, could still be regarded as clinically manageable with the routine clinical remounting and occlusal adjustment procedures [33]. The extra-oral tracing method, though, gave the discrepancies of about 0.74 mm at centric and 0.86 mm at protrusive, which could translate into the level of clinical perceptible mistakes that needed correction of a larger scope [34, 35]. It should be admitted that the exploration of these results should be done with subtle clinical judgment. Although the Nick and Notch method yields the best records in this research, it is very much dependent on the ability of the clinician to direct the mandible and get the patient to cooperate with the neuromuscular system. Graphic tracing techniques can have an advantage particularly in patients with impaired neuromuscular control, habitual protrusive closure, or more ridge resorption because of visual confirmation of the centric position at the apex of the Gothic arch design [36]. The choice of recording method must hence be personalized according to clinical conditions, patient factor and also according to the experience of the operator. There are a number of limitations of this research that should be taken into consideration. The twenty patients used as the sample size are enough to conclude that there are statistically significant differences but might be too limited to extend the results to the broader clinical populations. Age limit of 45-60 years was used to exclude the old patients who often report with the most difficult edentulous situations. This standardization through a single operator in all recordings brings about the possibility of operator biasing which might not represent variability as experienced in the normal clinical practice. Although the split- cast assessment is objective and quantitative, it determines discrepancy in one plane and might not accurately represent three-dimensional positional errors. The evidences of technique selection should be reinforced with future studies that will include larger samples, operators, three-dimensional analysis tools and relationship with clinical outcomes.

## Conclusion:

Accurate recording of centric and protrusive jaw relations remains a fundamental yet technique-sensitive step in complete denture prosthodontics. Among the evaluated methods, the Nick and Notch technique demonstrated superior consistency, while intraoral Gothic arch tracing proved more reliable than extraoral tracing, though none of the techniques were entirely free from error. Therefore, the selection of a jaw relation recording method should be individualized, balancing accuracy, clinical feasibility and patient-specific factors to achieve optimal prosthodontic outcomes.

## References

[1] Potdukhe SS (2023). J Indian Prosthodont Soc..

[2] Zhou TF (2023). Beijing Da Xue Xue Bao Yi Xue Ban..

[3] Gheedle R, John P (2023). J Contemp Dent Pract..

[4] Kumar CD (2023). Ann Afr Med..

[5] Keerthana SR (2021). J Indian Prosthodont Soc..

[6] de Sousa Ervolino IC (2023). J Prosthodont..

[7] Das A (2021). J Prosthet Dent..

[8] de Moraes Melo Neto CL (2021). Gen Dent..

[9] Utz KH (2023). Int J Prosthodont..

[10] Singh K (2021). J Contemp Dent Pract..

[11] Lassmann L (2024). J Prosthet Dent..

[12] de Moraes Melo Neto CL (2022). Eur J Dent..

[13] Yang LY (2020). Hua Xi Kou Qiang Yi Xue Za Zhi..

[14] Mohamed SM, ElGhannam MMS (2025). J Prosthet Dent..

[15] Naqash TA (2020). Niger J Clin Pract..

[16] Wei X (2021). Case Rep Dent..

[17] Kattadiyil MT (2021). J Prosthodont..

[18] Li W (2022). J Oral Sci..

[19] Manshaee F (2023). Dent Res J (Isfahan)..

[20] Ñahuincopa-Ríos RJ (2021). Rev Cient Odontol (Lima)..

[21] Stafeev A (2020). Int J Environ Res Public Health..

[22] Sonnahalli NK (2022). J Oral Biol Craniofac Res..

[23] Orgev A (2024). Int J Prosthodont..

[24] Praveena K (2021). J Pharm Bioallied Sci..

[25] Parisini P (2024). J Prosthet Dent..

[26] He H (2023). BMC Oral Health..

[27] Potdukhe SS (2024). J Indian Prosthodont Soc..

[28] Cheng CH (2022). Healthcare (Basel)..

[29] Miao X (2025). J Prosthet Dent..

[30] Choi H, Kang S (2022). J Yeungnam Med Sci..

[31] Cheng CH (2022). Healthcare (Basel)..

[32] Aljohani AO (2023). Life (Basel)..

[33] Zimmerer A (2021). Sci Rep..

[34] Ng LS (2022). F1000Res..

[35] Park H (2024). J Korean Assoc Oral Maxillofac Surg..

[36] Sushma R (2019). J Indian Prosthodont Soc..

